# Use of a Social Robot (LOVOT) for Persons With Dementia: Exploratory Study

**DOI:** 10.2196/36505

**Published:** 2022-08-01

**Authors:** Birthe Dinesen, Helle Kidde Hansen, Gry Bruun Grønborg, Anne-Kirstine Dyrvig, Sofie Dalskov Leisted, Henrik Stenstrup, Cathrine Skov Schacksen, Claus Oestergaard

**Affiliations:** 1 Laboratory for Welfare Technologies - Digital Health & Rehabilitation, Sports Sciences - Performance and Technology Department for Health Science and Technology Aalborg University Aalborg Denmark; 2 The Danish Clinical Quality Program, National Clinical Registries Odense Denmark; 3 The Innovation Department for the Elderly Aalborg Municipality Aalborg Denmark

**Keywords:** dementia, social robots, artificial intelligence, health care professionals, health care, persons with dementia

## Abstract

**Background:**

Approximately 50 million people worldwide are living with dementia. Social robots have been developed and tested to determine whether they improve the quality of life for persons with dementia. A new mobile social robot called LOVOT has artificial intelligence and sensor technologies built in. LOVOT, which is manufactured in Japan, has not yet been tested for use by persons with dementia.

**Objective:**

This study aimed to explore how the social robot LOVOT interacts with persons with dementia and how health care professionals experience working with LOVOT in their interaction with persons with dementia.

**Methods:**

The study was carried out at 3 nursing homes in Denmark, all with specialized units for persons with dementia. The interaction between the persons with dementia and LOVOT was tested in both individual sessions for 4 weeks and group sessions for 12 weeks. A total of 42 persons were included in the study, of which 12 were allocated to the individual sessions. A triangulation of data collection techniques was used: the World Health Organization-5 questionnaire, face scale, participant observation, and semistructured focus group interviews with health care professionals (n=3).

**Results:**

There were no clinically significant changes in the well-being of the persons with dementia followed in the individual or group interaction sessions over time. The results from the face scale showed that in both the individual and group sessions, persons with dementia tended to express more positive facial expressions after the sessions. Findings on how persons with dementia experienced their interaction with LOVOT can be stated in terms of the following themes: LOVOT opens up communication and interaction; provides entertainment; creates a breathing space; is accepted and creates joy; induces feelings of care; can create an overstimulation of feelings; is not accepted; is perceived as an animal; is perceived as being nondemanding; and prevents touch deprivation. Findings regarding the health care professionals’ experiences using LOVOT were as follows: the artificial behavior seems natural; and it is a communication tool that can stimulate, create feelings of security, and open up communication. Our findings indicate that the social robot is a tool that can be used in interactions with persons with dementia.

**Conclusions:**

The LOVOT robot is the next generation of social robots with advanced artificial intelligence. The vast majority of persons with dementia accepted the social robot LOVOT. LOVOT had positive effects, opened up communication, and facilitated interpersonal interaction. Although LOVOT did not create noticeable effects on social well-being, it gave individual persons a respite from everyday life. Some residents were overstimulated by emotions after interacting with LOVOT. Health care professionals accepted the social robot and view LOVOT as a new tool in the work with persons with dementia.

## Introduction

Approximately 50 million people worldwide are living with dementia [1]. Dementia causes deterioration in memory and mental skills such as speech. Living with dementia in everyday life can affect the person’s mood, causing apathy, depression, and anxiety [1,2]. Dementia is a progressive disease, such that persons with a severe degree of dementia must live in nursing facilities that specialize in dementia care. With an increasing prevalence of dementia, health care professionals are being challenged to provide quality care and give optimum attention to persons living with dementia. New technological innovations, such as social robots, have been developed and tested to assess whether they could improve the quality of life for persons living with dementia [3]. Social robots are designed to interact with humans to increase social interaction and improve well-being [4]. Góngora Alonso et al [5] have elaborated a 4-way classification of social robots: pet robots, humanoid robots, telepresence robots, and socially assistive robots (SAR). Examples of currently deployed social robots are PARO (robot seal), Aibo (robot dog), NeCoRo (robot cat), and CuDDler (robot teddy bear) [6].

A review by Góngora Alonos et al [5] concluded that the use of social robots for persons with dementia helped provide security and reduce stress. A systematic review of the use of social robots in mental health and well-being found that SAR are used largely with persons with dementia. However, these are only pilot studies, and there are limitations in the methods applied [3].

A review by Ghafurian et al [7] showed that social robots for the care of persons with dementia have received the most attention in the literature in the context of therapy or for increasing engagement, whereas robots designed for assisting with daily activities or providing health guidance received relatively limited attention. PARO was the most commonly used robot in dementia care studies [7]. A review of the use of PARO for persons with dementia has identified benefits such as improved mood, improved social engagement, and reduced negative emotions [8]. PARO’s ability to positively influence mood is indicated by the person with dementia becoming more active and relaxed and smiling. In addition, PARO has been shown to improve both verbal and visual engagement in social interactions. Factors that inhibited the use of PARO were the cost of the robot, increased workload for health care professionals working with the robot, infection concerns, and stigma and ethical issues related to a social robot in dementia care [6].

Despite the potential benefits of social robots for persons with dementia, the use of social robots currently faces several challenges. The current evidence base assessing the benefits of social robots for persons with dementia is still at an early stage, with relatively few studies. In addition, many of the existing study methods are characterized by short-term intervention durations and only a few subjects enrolled in the trials [5,9]. Furthermore, a lack of acceptance or outright resistance to social robots among older persons or those living with dementia has also been identified. This resistance may be explained by the fact that the robots studied so far have had limited social and auditory abilities; as such, they were unable to respond to any emotion or react to persons with dementia, nor were they fully aware of the social context [7,10,11].

**Figure 1:**
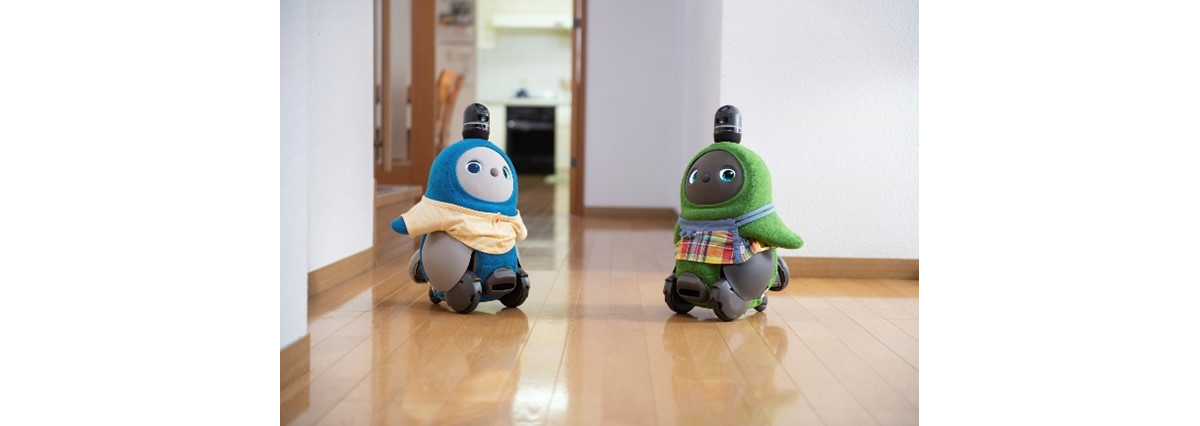
LOVOT as a social robot (reproduced from Groove X [12], with permission from Groove X).

Some of these deficiencies have been alleviated by the development of a new mobile social robot called LOVOT, which is manufactured in Japan. LOVOT possesses artificial intelligence and sophisticated sensor technologies. LOVOT has its own personality that develops over time with the purpose of creating joy in the user or patient [12]. Until our study, LOVOT had not yet been tested among persons with dementia. Figure 1 shows a photo of 2 LOVOT robots.

This study aims to explore (1) how the social robot LOVOT interacts with persons with dementia who are living in nursing homes in Denmark; and (2) how health care professionals experience working with LOVOT in their everyday interaction with persons with dementia.

## Methods

### LOVOT Specifications

**Figure 2:**
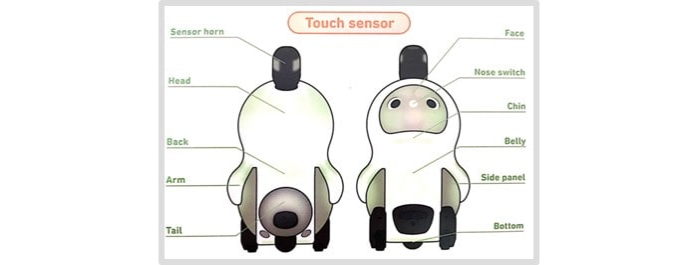
Anatomy of LOVOT (reproduced from Groove X [12], with permission from Groove X).

The social robot LOVOT was developed by the Groove X company. LOVOT weighs 4.2 kg and has a width of 28 cm, a height of 43 cm, and a depth of 26 cm. LOVOT is built with artificial intelligence, which makes it move in real time and act like a human being. LOVOT uses multiple sensors all over its body, including touch and distance sensors. The touch sensors are used to make LOVOT recognize stimulations on the body and can be warm or cold; it can even “fall asleep” when a person is stimulating the sensors. Distance sensors are used to determine the distance to objects, which makes it possible for LOVOT to move around without colliding with objects or walls [12]. The anatomy of LOVOT can be seen in Figure 2 and Multimedia Appendix 1. LOVOT was designed with a block-shaped “horn” on top of the head, as shown in Figure 2.

The horn can be used to turn LOVOT on and off. The horn includes a 360-degree thermal camera, which is used to determine humans from objects. The horn can also determine the direction of sounds and voices. LOVOT is implemented with 3 wheels, which can be enabled whenever LOVOT wants to move. LOVOT can be active for 40 minutes, after which it will need to return to its charging nest for a 20-minute charge. LOVOT can normally find and connect with the nest itself, although sometimes it needs help locating the nest. LOVOTs are designed with different personalities. For example, it can be programmed to be shy or outgoing in its personality. The personality can change over time as it gets more familiar with its user. Groove X has also developed an app that can be used in collaboration with each LOVOT. The app enables LOVOT to access the internet. LOVOT can use the internet to navigate over distances. LOVOT can take pictures of faces and use facial recognition as part of its artificial intelligence. In this study, LOVOT was not connected to the internet due to the European General Data Protection Regulation [13] and to avoid data being stored on a foreign server at Groove X in Japan [12].

### Ethical Considerations

The North Denmark Region Committee on Health Research Ethics was contacted to ensure that the project would be approved by the Ethical Committee. As the project did not include a new treatment approach and because the social robot LOVOT was not a medical device, the project did not require approval by the Ethical Committee (according to mail correspondence on September 23, 2019). Nevertheless, we have followed the Helsinki Declaration. Some persons with dementia were capable of signing the informed consent themselves, but the majority of the older persons had their guardian (spouse, daughter, or son) sign the informed consent form on behalf of the person with dementia. A data sharing agreement has been signed between the parties of the project.

### Context and Intervention of the Study

The study took place at 3 nursing homes in Denmark, all with specialized units for persons with dementia. The nursing homes were located in the Danish Municipalities of Aalborg, Viborg, and Skive.

The interaction between the persons with dementia and LOVOT was tested in both individual and group sessions. Individual interaction sessions between the person with dementia and the LOVOT robot took place over a 4-week period. The aim of the interaction sessions was to facilitate the activities of daily living (eg, eating and getting out of bed), companionship, health guidance (eg, receiving vaccinations), and individual engagement (eg, receiving visits from a relative) between the person with dementia and LOVOT. The individual sessions were facilitated by a health care professional and included approximately two 20- to 30-minute sessions with LOVOT per week. Group sessions, where a group of 4 to 6 persons interacted with 2 robots, were held over a 12-week period. The aim of these sessions was to facilitate communication and interaction between the persons and LOVOTs. Each of the 3 participating nursing homes established 2 groups. The group sessions, facilitated by a health care professional, lasted from 30 to 45 minutes and were held twice a week.

### Participants and Recruitment

The participants in the study were recruited based on the inclusion and exclusion criteria listed in Textbox 1.

Participants enrolled in the study were diagnosed with dementia prior to the study and before they moved into the specialized nursing homes for persons with dementia. In Denmark, persons are diagnosed with dementia at the geriatric ward of a hospital and in collaboration with the person’s own general practitioner via memory test, blood samples, computer tomography scan of the brain, and the assessment of the person’s daily functioning in everyday life. The researchers were not involved in this assessment process.

Before recruiting the persons for the LOVOT study, we conducted meetings in the specialized nursing homes with persons with dementia, their relatives, and health care professionals. The aim of the meetings was to introduce LOVOT and its functions and give further information about the trial. At the meetings, participants were able to ask questions about the trial.

**Figure 3:**
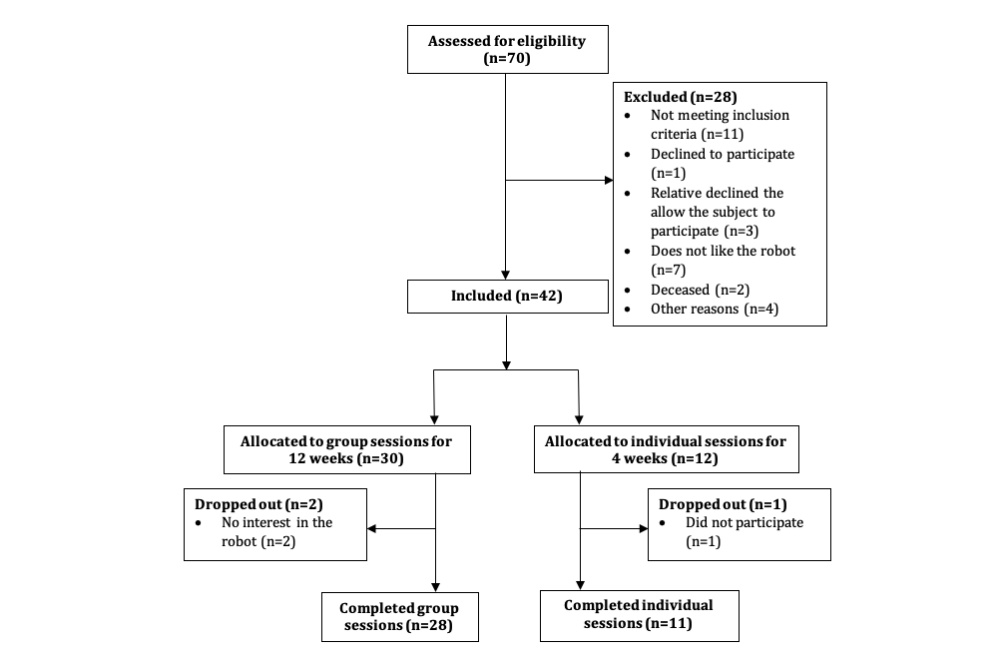
CONSORT (Consolidated Standards of Reporting Trials) diagram of the number of older adults included in this study.

Figure 3 shows a CONSORT (Consolidated Standards of Reporting Trials) diagram of the included persons and number of persons completing the sessions.

**Inclusion and exclusion criteria for participation in the study.**

**Inclusion criteria**

- Diagnosed with mild dementia
- Lives at 1 of the participating nursing homes in Aalborg, Viborg, and Skive Municipalities
- Meets one or more of the following behavioral criteria:
  Lonely
  High arousal
  Introverted behavior

**Exclusion criteria**

- Refusal to participate
- Diagnosed with a neurological disorder
- Diagnosed with a psychiatric disorder

### Outcome Measures and Data Collection Techniques

The outcome measures of the study were the following:

- Well-being
- Impact on the person’s mood
- Impact on the person’s behavior
- Acceptance of LOVOT
- LOVOT’s interaction with persons with dementia

Outcome measures, data collection techniques, and the time of collection for individual and group sessions are shown in Tables 1 and 2. The data collection process is described below.

**Table 1:** Overview of outcome measures and data collection techniques for the individual sessions.

Outcome measures	Data collection techniques	Baseline	Week 1	Week 2	Week 3	Week 4
Well-being	World Health Organiztion-5 questionnaire	✓		✓		✓
LOVOT’s impact on the person’s mood	Face scale		✓	✓	✓	✓
LOVOT’s impact on the person’s behavior	Participant observation		✓			✓
Acceptance of LOVOT	Participant observation		✓			✓
LOVOT’s interaction with the person	Focus group interviews with health care professionals	✓				✓

**Table 2:** Overview of outcome measures and data collection techniques for the group sessions.

Outcome measures	Data collection techniques	Baseline	Week 1	Weeks 2-12
Well-being	World Health Organization-5 questionnaire	✓		✓ (weeks 2, 4, 6, 8, 10, and12)
LOVOT’s impact on the person’s mood	Face scale		✓	✓ (weeks 2, 4, 6, 8, 10, and 12)
LOVOT’s impact on the person’s behavior	Participant observation		✓	✓ (weeks 4, 8, and 12)
Acceptance of LOVOT	Participant observation		✓	✓ (weeks 4, 8, and 12)
LOVOT’s interaction with the persons	Focus group interviews with health care professionals	✓		✓ (week 12)

### Well-Being

The World Health Organization-5 questionnaire (WHO-5) was used to measure the well-being of the persons over the course of the test period. The questionnaire consists of 5 questions, with responses scored from 1 to 5—a higher response score indicating greater well-being. The questionnaire was administered by the health care professionals and based on their perception of the person’s well-being. Since the health care professionals knew the persons very well, they were capable of making an informed assessment. The questionnaire was administered at baseline and then every other week during the test period for both the individual and the group sessions. The data analysis was performed according to WHO guidelines [14].

### Face Scale

The face scale [15] was used to measure LOVOT’s impact on the persons’ mood before and after a session. The original face scale, inspired by Wada et al [16], consists of 20 facial expressions. For our study, we selected the 7 most common expressions and set up a 7-point scoring scale, with 1 being the most positive expression and 7 being the most negative expression. The face scale was measured by the health care professionals once a week during the test period. The health care professionals knew the persons very well and were therefore capable of making an informed assessment. The data analysis was performed according to guidelines described by Lorish and Maisiak [15].

### Participant Observations

During the sessions, the health care professionals caried out observations [17] of the person’s behavior when interacting with LOVOT. We designed an observational guide [15] that focused on observations such as nonverbal behavior, interaction, and communication of the persons in their interaction with LOVOT. The health care professionals had received training in carrying out and recording their observations. By using the health care professionals as observers instead of outside researchers, we eliminated the risk that we would attract the person’s attention during their interaction with the robot. In addition, COVID-19 restrictions prevented researchers from entering the nursing homes. The observations were documented in a text file and analyzed by researchers using NVivo qualitative data analysis software (version 12.0; QSR International).

### Semistructured Focus Group Interviews

The 3 semistructured focus group interviews, inspired by Brinkmann and Kvale [18], were conducted with the health care professionals at each nursing home. The first interview was a baseline interview, which was to obtain knowledge about each person’s life history and dementia. The collection of data from each person included their age, gender, work history, family information, life history, and dementia symptoms. After the test period, we conducted the second, follow-up interview. The aim of the second interview was to explore how each person with dementia had interacted with LOVOT. Each interview lasted 60 to 90 minutes and was tape-recorded and transcribed. The interviews were coded using NVivo software and analyzed in steps inspired by Brinkmann and Kvale [18].

### Data Analysis

The quantitative data, collected using the face scale and WHO-5, were analyzed by calculating the median and IQR for the data from the individual and group sessions. The 5 questions from the WHO-5 were summarized and multiplied by 4 to generate a range of values between 0 and 100, with a score of 0 indicating the worst possible well-being and a score of 100 indicating the best possible well-being for the older person. A clinically significant change in the WHO-5 score is assessed if we recorded a change of at least 10%, corresponding to 10 points in WHO-5. Data are presented in graphs, showing the median and IQR. The interviews and observational notes were analyzed using NVivo software, in steps inspired by Brinkmann and Kvale [18].

## Results

### Baseline Data

A total of 42 persons with dementia were included in the study, of which 30 were allocated to the group sessions and 12 to the individual sessions. The sociodemographic and clinical characteristics of the participants in the individual and group sessions at baseline are shown in Table 3. We use either the number of persons and percentage or the median and IQR for the different parameters.

**Figure 4:**
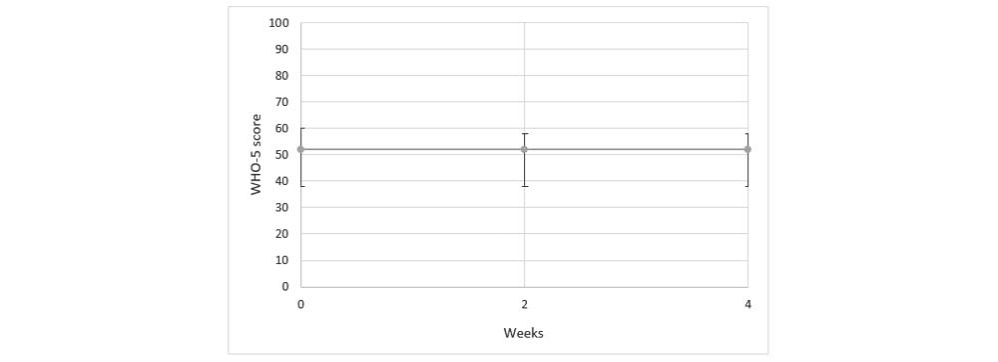
Results from individual sessions using the WHO-5 questionnaire. WHO: World Health Organization.

**Figure 5:**
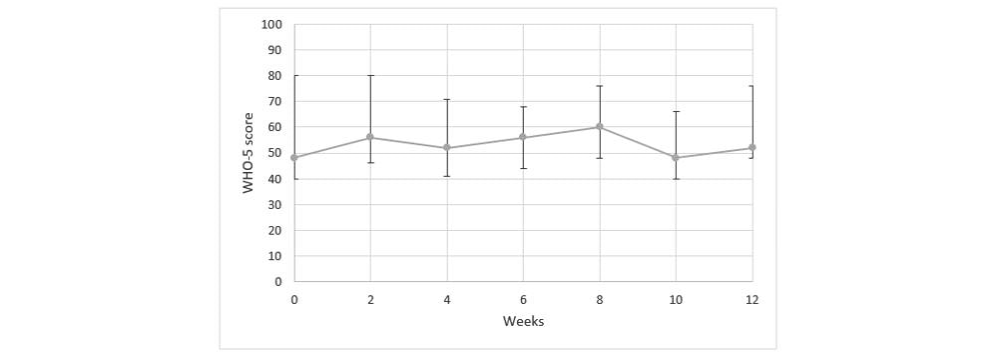
Results from the WHO-5 questionnaire for the group sessions over the 12-week test period. WHO: World Health Organization.

In Figure 4, the results from the individual sessions using the WHO-5 questionnaire are presented. In Figure 5, the results from groups sessions over a 12-week test period are presented.

**Table 3:** Characteristics of the persons at baseline.

Characteristic		Individual sessions (n=12)	Group sessions (n=30)
**Gender, n (%)**			
	Male	1 (8)	8 (27)
	Female	11 (92)	22 (73)
Age (year), median (IQR)		83 (67-92)	84 (66-96)
Years at nursing home, median (IQR)		1.75 (0.5-4)	1.9 (0.08-5)
Years with dementia, median (IQR)		2 (0.5-10)	3,5 (0.25-10)
Have children, n (%)		10 (83)	27 (90)
**Type of dementia, n (%)**			
	Alzheimer disease	8 (67)	12 (40)
	Other	4 (33)	18 (60)

### Impact on Mood

**Figure 6:**
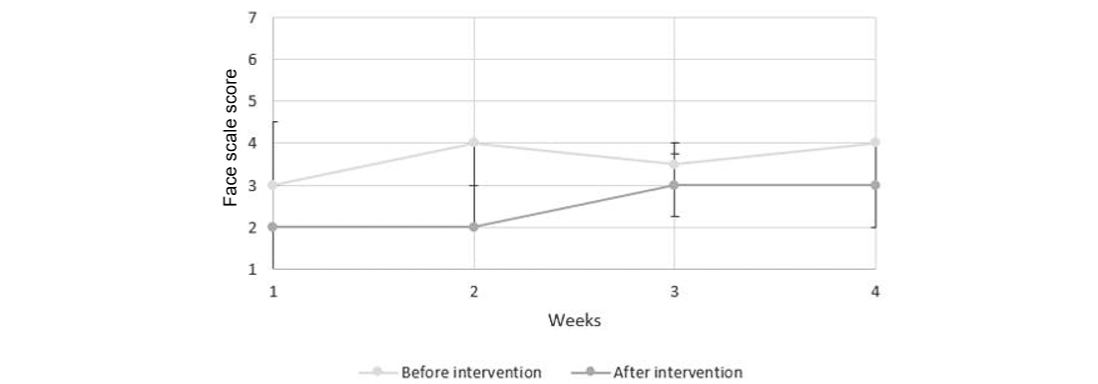
Results from individual sessions using the face scale.

Figure 6 presents the results attained from the face scale for the individual sessions. A higher score expresses a more negative face expression. Therefore, based on the median score illustrated in Figure 6, there is a trend toward more negative facial expressions before the LOVOT sessions than after the LOVOT sessions were completed.

**Figure 7:**
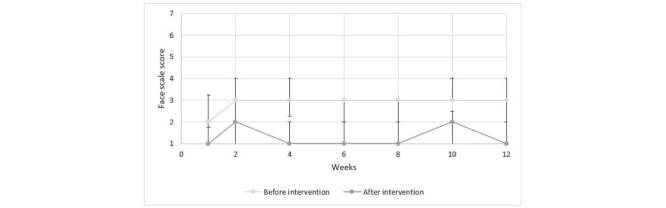
Results from group sessions using face scale.

Figure 7 presents the results attained from the face scale for the group sessions. Based on the median score illustrated in Figure 7, there is a trend toward more negative facial expression prior to undertaking the LOVOT sessions than after the LOVOT sessions were completed.

### Qualitative Findings

#### Focus Group Interviews

Tables 4 and 5 present findings on how the persons and health care professionals responded to LOVOT and the effect of LOVOT on the persons with dementia. These findings are based on the follow-up focus group interviews with the health care professionals and the professionals’ own observations at each session. In the following sections, the findings from the focus group interviews are supplemented with illustrative quotations taken from the focus group interviews. We consider these quotations to be representative of our findings.

**Table 4:** Findings on the experiences of how the persons experienced the interaction with LOVOT along with quotations from focus groups (FGs) with health care professionals from the nursing homes.

Theme/category	Illustrative quotation
Diverts and is calming	“This weekend, he also had a period where he wanted to go home, and during the shift change, I used LOVOT to calm him down.” (FG 1)
Opens up communication and interaction	“When LOVOT is there, she smiles and is happy. She speaks more with the other [older] persons. She’s someone who doesn’t say much.” (FG 1)
Provides entertainment	“They are fun to watch, both individually and together.” (FG 1)
Creates a breathing space	“LOVOT has given her a boost, a breathing space, where there is just something positive in her everyday life.” (FG 1)
Is accepted and creates feelings of happiness	“When she got LOVOT up in her hand, she started crying because she was so happy...it evoked feelings of happiness. She was moved to tears, absolutely.” (FG 1)
Induces feelings of care	“She has stepped into a mother role. She was one of the first ones we noticed who started treating it like a child. She sits and rocks it...She sits and rocks her leg just like you do with an infant or at least a little baby. She really just wants to sit with it and then just have that feeling.” (FG 2)
Can create an overstimulation of feelings	“He was quickly taken away because he reacted violently after being with LOVOT.” (FG 2)
Is not accepted	“She was not really able to relate to LOVOT. She has had other things in mind. She cannot find peace with it. She can just look at it and say, ‘Yes,’ but she has something else going on. So it has not had any positive effect on her either.” (FG 2)
Is perceived as an animal	“But she clearly sees it as something animal, because she is very fond of dogs, so she almost claps her hands when they come.” (FG 1)
Is perceived as being nondemanding	“But she has also always talked to it as if it were a person who was with her and has meant a lot. I don’t know whether a person with dementia can relate more to such a thing compared to us humans, because we demand something, I don’t know if they have that feeling. Because LOVOT demands nothing, [like] a dog, other than to be petted. The rest of us always demand something.” (FG 3)
Prevents “skin hunger”	“But we talked a little about touch deprivation...She sat with [LOVOT] on the sofa, where it sat up next to her, and she sat like that and cuddled it. She has received the warmth from LOVOT and the sounds. It can stimulate something in relation to skin hunger when she does not have much contact and touch with others.” (FG 2)

**Table 5:** Findings on how the health care professionals experienced using LOVOT along with quotations from focus groups (FGs) with health care professionals from the nursing homes.

Theme/category	Illustrative quotation
Artificial behavior appears natural	“LOVOT’s behavior seems natural, even though we know it is artificial intelligence, the robots have different personalities.” (FGs 1, 2, and 3)
Communication tool that can stimulate, create feelings of security, and facilitate communication	“We think that LOVOT is a good tool for creating space for collaboration with residents with dementia.” (FGs 1 and 2) “Relatives have also been very positive about what LOVOT is doing to her. Her daughter has also been to a session and seen what it does to her. Her eyes lit up so completely, and she smiled and became happy. And she spoke to [LOVOT] as if it were a child. She knows it’s a robot.” (FG 2)
LOVOT is user-friendly and fun	“It is user-friendly and easy to operate, but it’s difficult for a person with dementia.” (FGs 1, 2, and 3)

#### Persons With Dementia and Their Interaction With LOVOT

The health care professionals described LOVOT as having an entertaining and calming effect on the persons with dementia. LOVOT has also influenced the persons to communicate and interact more with each other and the staff. The health care professionals stated that LOVOT provided a degree of entertainment value for the persons. It was further described how the interaction with LOVOT allowed the person to have a breathing space and relax. According to the health care professionals, some of the persons accepted LOVOT, and it evoked positive feelings, even joy. LOVOT also evoked feelings of care for the persons, where some of the persons treated LOVOT as if it were a child. Being together with LOVOT was at times even overstimulating for some of the older persons. Not all the participants in the study accepted LOVOT. Some of the persons with dementia thought that LOVOT was simply nonsense, whereas others found the robot difficult to relate to or interact with. The staff also described how some of the persons perceived LOVOT as an animal and interacted with LOVOT as they might with an animal, such as snapping at it or calling LOVOT to get its attention. The health care professionals further described how this acceptance and promotion of positive feelings could be due to the residents’ perceiving LOVOT as not demanding anything other than being petted. The ability to touch and hug LOVOT has been shown to help prevent touch deprivation in some of the persons who might not have much physical contact with others.

#### Health Care Professionals’ Experience With LOVOT

The health care professionals described how LOVOT’s artificial behavior seemed natural. The health care professionals described LOVOT as an effective tool for communication between the staff and persons with dementia, in that it can create feelings of security and facilitate communication. LOVOT was described as user-friendly and fun, but the health care professionals stated that LOVOT could also be a burden on some of the residents.

## Discussion

### Principal Findings

Our results showed that there were no clinically significant changes in the well-being of the persons with dementia who participated in the individual or group sessions with the LOVOT robot. Results from the face scale showed that in both the individual and group sessions, the persons with dementia tended to express more positive facial expressions after the session with LOVOT. In other words, interacting with LOVOT made them happier. The effect on mood varied throughout the test period, however. The results indicated that LOVOT may have a positive impact on the current mood of the person with dementia, but this is not a sustained effect over time. Findings on how persons with dementia experienced their interaction with LOVOT can be summarized in terms of the following: the robot has an amusing or calming effect; facilitates more open communication and interaction; has some entertainment value; creates a breathing space; is accepted and creates a degree of happiness or good feeling; creates feelings of care; can even create an overstimulation of feelings at times; may not be accepted by all residents; can be perceived as an animal; is perceived as being nondemanding; and prevents touch deprivation. We emphasize that LOVOT was not intended as, nor did it prove to be, an effective tool for each person with dementia.

Findings on the health care professionals’ experiences using LOVOT indicated that they found that its artificial behavior seems natural; that LOVOT is viewed as a communication tool that can stimulate, create feelings of security, and facilitate communication; and that LOVOT is viewed as user-friendly, fun, and a positive tool. The health care professionals found that the social robot, as a new tool in their “care toolbox,” can be used in interactions with persons with dementia. The professionals expressed no ethical dilemmas regarding the use of the robot.

In our study, we did not identify any significant changes in the well-being of persons with dementia during the period when they had interactions with the social robot. We think that the persons did not have sufficient time for interactions with LOVOT in the individual and group sessions, such that insufficient time for adjustment might explain the lack of any identified changes in their well-being.

The finding that LOVOT can have a positive impact on the person’s current mood is consistent with the results of other studies that have examined the effects of social robots on persons with dementia. Wada et al [16] also used the face scales to evaluate the influence of the robot seal PARO on the mood of the persons, studying their interaction over a 3-month period. Wada et al [16] found that the face scale score was lower after the session than before the start of the session. A systematic literature review by Kang et al [19] describes a study that examined persons’ facial expressions during group sessions with PARO over a 6-week period. Here, significant positive changes were noted: following their interactions with PARO, the persons were smiling more and happier. Both these studies are limited by their low sample size of the persons, but they nevertheless support the findings of LOVOT’s positive impact on the persons’ momentary mood.

The LOVOT robot is the next generation of social robots with advanced artificial intelligence. The LOVOT has not previously been tested in any clinical settings outside of Japan. The social robot LOVOT is still under development and can be categorized as the most advanced SAR at the moment internationally. We have not identified other studies that have documented these findings, as this is the first study that tests LOVOT interacting with persons with dementia. However, studies of other, less advanced social robots interacting with persons with dementia found that social robots can provide positive outcomes; they can improve social engagement, such as facilitating more communication and promoting positive mood [6-8]. We found that LOVOT was able to open up communication and enhance the expression of feelings, laughter, and feelings of care due to LOVOT’s advanced ability to respond and interact with human beings in a humanlike way. LOVOT’s state-of-the-art artificial intelligence gave it a certain advantage here. A systematic review and meta-analysis of randomized controlled studies by Pu et al [20] found that social robots appeared to reduce agitation and anxiety and enhance the quality of life for older adults, but the studies were not statistically significant. A narrative review by Pu et al [20] indicated that social robots can improve engagement, enhance interaction, reduce loneliness, and reduce stress indicators.

We found that LOVOT could create an overstimulation of feelings for persons with dementia. Participant-observation notes showed that some persons were either crying or became extremely extroverted in their behavior. Robinson et al [10] have found that some persons with dementia may find that the behavior of some social robots provokes anxiety. A systematic review by Hung et al [8] on the use on PARO in care settings found that PARO could cause negative emotional responses, including fearfulness, anger, and agitation. In their review, Hung et al [8] question whether past negative experiences with animals could have influenced whether the person “likes” or “dislikes” a social robot. Further research is needed to explore this variable.

Some persons with dementia in our study did not accept the LOVOT. Our consort diagram shows that 7 persons out of the 70 accessed for eligibility did not like the robot, equivalent to 10% of our sample. As social robots are new to dementia care, it is understandable that not everyone in the older generation would be comfortable accepting the LOVOT. The range of attitudes about social robots is also confirmed by other studies [5,6,20].

Health care professionals felt that LOVOT’s artificial behavior seemed natural in its interactions with the persons with dementia, and overall, they found LOVOT to be an effective tool for communication and interaction for persons with dementia. The review by Hung et al [8] found that the use of social robots in dementia care can lead to perceptions that care has become infantilizing and dehumanizing. However, this aspect was not found in our study. One may question if this perception is due to the appearance of LOVOT and its potential to interact with persons with dementia. This issue needs to be explored further in future international studies. Abdi et al [6], in a scoping review on the use of SAR in care for older persons, identified several potential roles that the SAR could have—as affective therapy, cognitive training, social facilitator, companionship, and physiological therapy. Ghafurian et al [7] have emphasized the need for more robust research, in an international context, to fully assess the value of SAR in care for older persons. As social robots become more advanced, with artificial intelligence, there is a need for further, comprehensive testing of social robots within care for older persons and to develop a range of data collection techniques that can effectively assess the efficacy of social robots and identify the ethical issues connected with using social robots with persons living with dementia.

### Limitations

The target group for this trial was older persons with dementia who live in nursing homes. A limitation of the study is the gender distribution, as our sample had only 9 men among the 42 subjects. Another limitation is the fact that we were not able to interview the residents directly about their condition but had to rely on observations and data from health care staff. We have instead used a triangulation of data collection techniques to explore how the persons with dementia in our study (and the health care staff) experienced their encounter with LOVOT. However, it was the health care professionals who filled out the questionnaires about the residents. This had an advantage because the staff had intimate knowledge of each resident. Another limitation is that we have not incorporated the perspectives of the residents’ relatives in this study, which could have enriched our data regarding the use of SAR in dementia care for older persons. We are aware that this is a pilot study, and there will be a need to conduct studies with LOVOT using a longer duration period and on a larger scale to fully explore LOVOT’s potential and limitations for persons with dementia.

### Conclusions

The LOVOT robot is the next generation of social robots with advanced artificial intelligence. The vast majority of persons with dementia accepted the social robot LOVOT. LOVOT had positive effects, opened up communication, and facilitated interpersonal interaction. Although LOVOT did not create noticeable effects on social well-being, it gave individual persons a respite from everyday life. Some residents were overstimulated by emotions after interacting with LOVOT. Health care professionals accepted the social robot and view the LOVOT as a new tool in the work with persons with dementia. As social robots become more advanced, with artificial intelligence, there is a need for testing the advanced social robots within care for older persons and to develop a new toolbox that can fully assess the value of the social robots for persons with dementia in the health care sector.

## Appendix

Multimedia Appendix 1 Visualization of LOVOT’s anatomy (reproduced from Groove X [12], with permission from Groove X).

## Abbreviations

CONSORT: Consolidated Standards of Reporting Trials
SAR: socially assistive robots
WHO-5: World Health Organization-5 questionnaire
