# Supplementary figures and images for: Use of a Social Robot (LOVOT) for Persons With Dementia: Exploratory Study
Source: JMIR Rehabil Assist Technol. 2022 Aug 1;9(3):e36505. doi: 10.2196/36505 (PMC9379791; doi:10.2196/36505)

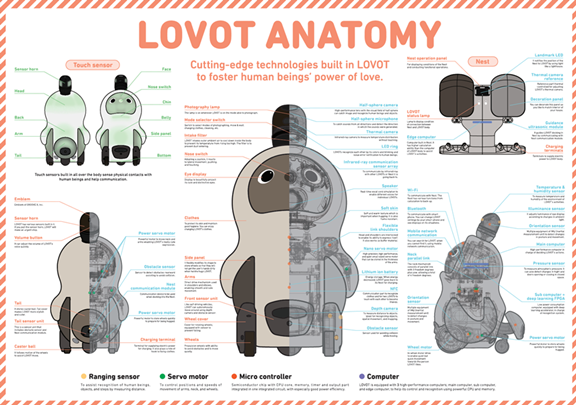

Supplement: Multimedia Appendix 1 [file rehab_v9i3e36505_app1.png]
